# Measuring Skill Growth and Evaluating Change: Unconditional and Conditional Approaches to Latent Growth Cognitive Diagnostic Models

**DOI:** 10.3389/fpsyg.2020.02205

**Published:** 2020-09-11

**Authors:** Qiao Lin, Kuan Xing, Yoon Soo Park

**Affiliations:** ^1^Department of Educational Psychology, University of Illinois at Chicago, Chicago, IL, United States; ^2^University of Tennessee Health Science Center, Memphis, TN, United States; ^3^Department of Medical Education, University of Illinois at Chicago, Chicago, IL, United States

**Keywords:** cognitive diagnostic model, covariate extension, latent growth curve, longitudinal analysis, learning progression

## Abstract

During the past decade, cognitive diagnostic models (CDMs) have become prevalent in providing diagnostic information for learning. Cognitive diagnostic models have generally focused on single cross-sectional time points. However, longitudinal assessments have been commonly used in education to assess students’ learning progress as well as evaluating intervention effects. Thus, it becomes natural to identify longitudinal growth in skills profiles mastery, which can yield meaningful inferences on learning. This study proposes longitudinal CDMs that incorporate latent growth curve modeling and covariate extensions, with the aim to measure the growth of skills mastery and to evaluate attribute-level intervention effects over time. Using real-world data, this study demonstrates applications of unconditional and conditional latent growth CDMs. Simulation studies show stable parameter recovery and classification of latent classes for different sample sizes. These findings suggest that building on the well-established growth modeling frameworks, applications of covariate-based longitudinal CDM can help understand the effect of explanatory factors and intervention on the change of attribute mastery.

## Introduction

Growth of knowledge and skills are important indicators of learning, which commonly results from the implementation of interventions such as course materials, instructional curriculum, teaching methods, and policies. For educational systems and educators, it is important to understand the changes in learning by evaluating the intervention effects. To quantify these effects, longitudinal assessments or pre-and post-test designs have been widely used and the raw score has been examined to reveal the progress in learning. Longitudinal assessment designs involve repeated observations of variables over a period of time while pre-and post-test designs focus on two measurements that are taken before and after a treatment. However, simple comparison between time points may lack reliability and validity (Linn and Slinde, 1977); therefore, researchers may seek applying psychometric models to measure students’ knowledge and aptitude that are characterized as latent constructs. Item response theory (IRT) allows psychometrically specifying students’ ability as continues latent variables and has a tradition to employ longitudinal models to assess the growth in ability (Andersen, 1985; Fischer, 1989; Embretson, 1991). The multidimensional IRT models are useful to measure how the unidimensional ability increase over a period of time, yet it is hard to diagnose the increment when the latent constructs are correlated with one another over repeated measures.

In recent years, cognitive diagnostic models (CDMs), also known as diagnostic classification models (DCMs), have drawn increasing attention from researchers, as provides diagnostic information for learning and instruction (Bradshaw and Levy, 2019). Several CDMs and assessments have been developed to evaluate examinees’ mastery status on a set of cognitive skills (e.g., DiBello et al., 1995; Bradshaw et al., 2014; Culpepper, 2019; Culpepper and Chen, 2019). Most commonly, CDMs have been used to assess students’ skills profiles at a single time point rather than measuring changes in skills proficiency over time. However, it is important for educators to know the students’ learning trajectories to achieve learning goals as well as the effects of intervention on the growth of student skills.

In this regard, latent transition analysis (LTA; Collins and Wugalter, 1992) has been incorporated to the recent development of CDM to evaluate changes in skills mastery. For example, Li et al. (2016) employed DINA model as the measurement model in an LTA to demonstrate a means of analyzing change in cognitive skills over time. Similarly, Kaya and Leite (2017) developed a model combining the LTA and deterministic input noisy “and” gate (DINA; Junker and Sijtsma, 2001) and deterministic input noisy “or” gate (DINO; Templin and Henson, 2006) CDMs to address within-individual and between-groups change in follow-up measurements of learning. In addition, Madison and Bradshaw (2018a) proposed the Transition Diagnostic Classification Model (TDCM) that combined log-linear cognitive diagnosis model (LCDM; Henson et al., 2009) and with LTA to provide a more general framework for measuring growth in cognitive diagnostic modeling. Compared to the models proposed by Li et al. (2016) and Kaya and Leite (2017) that assume specific item response structures and place constraints on parameters, TCDM use a general DCM framework that subsume early models and combine it with LTA. They further extended the TDCM to model multiple groups (MG-TDCM), thereby enabling the examination of group differential growth in attribute mastery in pre-and posttest design (Madison and Bradshaw, 2018b).

To model the learning trajectory, Wang et al. (2018) proposed a family of learning models that use higher-order, hidden Markov model (HO-HMM) to model attribute transition and incorporate CDM framework to understand individualized learning trajectory. Furthermore, Chen et al. (2018) proposed a class of dynamic CDM models to trace learning trajectories. They focused on investigating different types of learning trajectories and developed a Bayesian Modeling framework to estimate these learning trajectories. Focusing on modeling the growth in the higher-order latent trait, Lee (2017) proposed longitudinal growth curve cognitive diagnosis models (GC-CDM) that trace changes in the higher-order latent traits to incorporate learning over time into the cognitive assessment framework. Likewise, Huang (2017) embedded a multilevel structure into higher order latent traits and extended the generalized deterministic input, noisy “and” gate (G-DINA) mode to a multilevel higher order CDM, which enable the measurement of changes in the latent trait in longitudinal data. Most recently, Zhan et al. (2019) proposed a longitudinal diagnostic classification modeling approach for assessing learning growth in both repeated measures design and anchor-item design. Different from the LTA-based methods providing attribute-level transition probability matrix, the proposed longitudinal DINA model (Long-DINA) is able to provide quantitative values of overall and individual growth.

Although various longitudinal CDMs have been developed to measure the transition of examinees’ attribute mastery statuses over time, fewer studies focus on the intervention effects that drive the changes in skill mastery from the perspectives of covariates extension and latent growth curve model. In this research study, we proposed two latent growth CDMs by using unconditional and conditional approaches to trace changes in latent attributes over time as well as allowing a flexible parameterization to specify covariates that can be meaningful in studying a longitudinal data structure.

Different from other longitudinal CDMs in the literature, the latent growth CDMs proposed in this study is motivated by the well-established growth curve modeling framework that are commonly used in social sciences to measure latent growth (e.g., Duncan et al., 2013). And as such, it becomes important to link the longitudinal CDM framework to existing techniques in the social sciences, to prompt more generalizable and flexible model extensions. Although some previous studies have incorporated growth curve model into CDM, they mainly focused on extending the higher-order latent trait to model the growth in learning, which assumes associations between different latent attributes, that the probability of having the skills depends on a higher order overall ability. Moreover, a focus on attribute-level changes and their impact over time can be handled and specified in the latent growth CDM framework, as proposed in this paper. In this study, we incorporate covariate extensions and latent growth curve model into the attribute-level of CDM framework instead of the higher-order latent trait level. In this way, we can monitor changes in the attribute level directly under the independence assumption for attributes probabilities.

The study consists of three parts: A real-world data analysis and two simulation studies. We first demonstrate the model application using real-world data to motivate the rationale for the latent growth framework and to monitor changes in students’ skills mastery and intervention effects. The ensuing sections include two simulation studies conducted separately to examine the parameter recovery of the proposed models. In this manner, the simulation studies with varying longitudinal design components provide comprehensive inference for different number of time points, sample sizes, and covariate specification conditions. Findings from this study could help researchers apply the latent growth CDMs in practice and promote the development of longitudinal CDMs.

## Cognitive Diagnostic Models

Cognitive diagnostic models were designed to classify examinees into skill profiles that indicate their mastery in fine-grained skills or attributes based on their performance on a set of items (Rupp et al., 2010). It refers to a class of psychometric models where patterns of attributes mastery have been represented as latent classes. Distinguish from IRT models that latent traits are continuous, CDMs examine categorical latent traits. Different kinds of CDMs have been developed in literature and various generalizations of CDMs have also been proposed including the LCDM (Henson et al., 2009), general diagnostic model (GDM; von Davier, 2008), and the generalized DINA model (G-DINA; de la Torre, 2011).

### Reparameterized DINA (RDINA) Model

In this study, we use the DINA model to demonstrate the framework, which can be applied to other CDM families and generalizations of DINA models (e.g., von Davier, 2008; de la Torre, 2011). The DINA model is developed with the idea that in order to answer an item *j* correctly, the examinee *i* must have mastered all of the required skills (Tatsuoka, 1985). The binary latent variable η=i⁢j∏k=1kαi⁢kqi⁢k indicate whether the *i*th examinee has mastered the set of attributes α [i.e., attributes, α = (α_1,_ α_2_, _…_, α_*k*,_)′] to solve the *j*th item, where η_*ij*_ = 1 means the presence of the necessary set of attributes, and η_*ij*_ = 0 otherwise. As specified by the Q-matrix, *q*_*ik*_ is either zero or one, indicating whether the attribute *k* is required for solving item *j*. This study uses the reparameterized deterministic inputs noisy “and” gate (DeCarlo, 2011) to apply the longitudinal framework, as it facilitates incorporating covariates as intervention effects in latent growth curve model. The RDINA takes the logit of the traditional DINA model

(1) $$\text{logit}p\left(Y_{i\,j}=1|\eta_{i\,j}\right)=f_{j}+d_{j}\eta_{i\,j}$$

The *f*_*j*_ parameter indicates the log odds of a false alarm that is the probability of getting item *j* correct given the examinee do not have the requisite skills. The parameter *dj* provides a measure of how well the item can discriminate an examinee with or without the mastery of required skills. The guessing and slip parameters used in the DINA model can be recovered by exponentiating the RDINA parameters (DeCarlo, 2011)as:

(1.1) $$g_{j}=\text{exp}\,\left(f_{j}\right)/\left[1+\text{exp}\,\left(f_{j}\right)\right]$$

(1.2) $$s_{j}=1-\exp\left(f_{j}+d_{j}\right)/\left[1+\exp\left(f_{j}+d_{j}\right)\right]$$

### Covariate Extension to the RDINA Model

Various latent class models have incorporated covariates as extensions (Dayton and Macready, 1988; DeCarlo, unpublished). In the DINA model, covariates can be specified either at the item level and/or attribute level. Park and Lee (2014) proposed a covariate extension to the RDINA model by applying a latent class regression framework. In particular, when a discrete or a continuous covariate, $\underline{Z}$, is introduced into a latent class model, an examinee’s response probability can be expressed as

(2) $$p\,\left(Y_{i\,1},Y_{i\,2},\ldots ,Y_{i\,j}|\underline{Z}\right)=\sum_{\underline{\alpha }}p\,\left(\underline{\alpha }|\underline{Z}\right)\,\prod_{j}p\,\left(Y_{i\,j}|\underline{\alpha },\underline{Z}\right)$$

where $p\,\left(Y_{i\,1},Y_{i\,2},\ldots ,Y_{i\,j}|\underline{Z}\right)$ represents response probability conditioning on covariate $\underline{Z}$, $p\,\left(\underline{\alpha }|\underline{Z}\right)$ represents the covariate affecting the attribute probability, *p(Y_ij_*|α, $\underline{Z})$ represents the covariate affecting the response probability. In particular, the effects of the covariate on the response probability and attribute probability are shown as following:

(3) $$\text{logit}\,p\,\left(Y_{i\,j}|\alpha ,\underline{Z}\right)=f_{j}+d_{j}\,\eta_{i\,j}+l_{j}\,\underline{Z}$$

(4) $$\text{logit}\,p\,\left(\alpha_{k}|\underline{Z}\right)=b_{k}+h_{k}\,\underline{Z}$$

where the parameter *l*_*j*_ reflects the changes in the guessing and slip parameter for a unit change in $\underline{Z}$. Similarly, the parameter *h*_*k*_ indicates the changes in the attribute difficulty parameter (*b*_*k*_), when the covariate $\underline{Z}$ is conditional on the attribute level.

### Relationship Between RDINA and General Diagnostic Model

The RDINA model was employed in this study to establish the framework; however, the parameterization used in its covariate extension can be extended and reparameterized as special cases of the GDM (von Davier, 2005; Park and Lee, 2019). In the GDM, the observed response *X* is modeled for *i* items, x response categories, and *j* respondents as follows:

$$P\left(X=x|i,j\right)=\text{exp}\left[f\left(\lambda_{x\,i},\theta_{j}\right)\right]/\left\{1+\sum_{m}\text{exp}\left[f\left(\lambda_{x\,i},\theta_{j}\right)\right]\right\}$$

GDM item parameters are the λ_*xi*_ = (β_*xi*,_**q**_i_,γ_*xi*_), which include slope parameters and the Q-matrix specification, _*q***i**_. In the DINA where attributes are binary, the skill vector for examinee *j*, θ_*j*_ = (α_*j*1_,…,α_*jk*_), are binary values. As shown in von Davier (2014, p. 58), the DINA can be parameterized as a special case of the GDM as follows:

(6) $$P\left(X_{v\,i}=1|{\text{q}}_{i,}^{*},{\text{a}}^{*}\right)=\frac{\text{exp}\,\left(\beta_{i}+\sum_{k}\gamma_{i\,k}\,a_{k}^{*}\,q_{i\,k}^{*}\right)}{1+\text{exp}\,\left(\beta_{i}+\sum_{k}\gamma_{i\,k}\,a_{k}^{*}\,q_{i\,k}^{*}\right)}$$

When a covariate Z introduced to Eq. 5, the following *h*_*k*_ parameters are added:

(7) $$P\left(X_{v\,i}=1|{\text{q}}_{i,}^{*},{\text{a}}^{*},Z\right)=\frac{\text{exp}\,\left(\beta_{i}+\sum_{k}\gamma_{i\,k}\,a_{k}^{*}\,q_{i\,k}^{*}+h_{k}\,Z\right)}{1+\text{exp}\,\left(\beta_{i}+\sum_{k}\gamma_{i\,k}\,a_{k}^{*}\,q_{i\,k}^{*}+h_{k}\,Z\right)}$$

Taking the logit simplifies the model to the item-level of covariate extension approach as presented in Eq. 4.

### Latent Growth Curve Model

Latent growth curve model has been widely used in longitudinal analysis to estimate growth over time, such as examining the treatment effects in the pre-post intervention study. As a special case of structural equation model (SEM), latent growth modeling formwork extend SEM to represent repeated measures of dependent variables as a function of time and other measures. Based on the research of Tucker (1958), Rao (1958), Meredith and Tisak (1984) and Meredith and Tisak (1990) furthered SEM to model the interindividual differences in change. To model the changes in a variable over time, latent growth curve model assumes that there is a systematic trajectory of change underlying the repeated measures of the variable. In particular, for *i* (*i* = 1,2,…,*n*) subjects measured at *j* (*j* = 1,2,…,*t*) occasions, the measurement model of latent growth curve model can be expressed as

(8) $$y_{i\,j}=\lambda_{0\,j}\,\eta_{0\,i}+\lambda_{1\,j}\,\eta_{1\,i}+\varepsilon_{i\,j}$$

where *y*_*ij*_ is the outcome variable for individual *i* at time *j*. η_*0i*_ and η_*1i*_ represent latent trajectory parameters: individual’s initial level (i.e., intercept) and rate of change over time (i.e. slopes). ε_*ij*_ represent time-specific error for person *i.* In the structural model of latent growth curve model, these latent trajectory parameters become outcome variables that can be expressed as:

(8.1) $$\eta_{0\,i}=\mu_{0}+e_{0\,i}$$

(8.2) $$\eta_{1\,i}=\mu_{1}+e_{1\,j}$$

where μ_*0*_ represents the sample mean initial level, *e*_*0i*_ represents the deviations from mean initial level for individual *i*; μ_*1*_ represents the sample mean rate of change, *e*_*1i*_ represents the deviations from mean rate of change for individual *i*.

In latent growth curve model, λ_*0j*_ is fixed at 1 for all *j* occasions. It should be noted that the equations presented above are considered as an unconditional latent growth curve model because there is no covariate involved. A conditional latent growth curve model that contains covariates can be specified by adding predictors of the outcome variable into Eq 8. The corresponding covariates effects on the latent trajectory parameters could be included in Eqs 8.1 and 8.2.

### The Latent Growth Cognitive Diagnostic Model

Motivated from latent growth curve models and RDINA model with covariate extensions, we propose two latent growth curve CDMs (LG-CDMs) using unconditional and conditional approaches to track the changes in examinees’ latent attributes as well as evaluating the effects of covariate at the latent attributes level. For the LG-CDMs, an examinee *i*’s response probability *p*(*Y*_*ij*_ = 1|η_*ij*_) was specified by the RDINA model, which was shown in the above Eq. 1.

#### Unconditional LG-CDM

Motivated from the unconditional latent growth curve model with random intercept and random slope, we develop an Unconditional LG-CDM that includes unconditional latent growth curve to the attribute level of CDM framework as linear model. At the attribute level α_*k*_ = (α_1,_ α_2_, _…_, α_*k*,_)′, we assume a linear relationship between time and attributes to model the changes in attributes over time. When no covariate is specified, Eq. (9) shows the latent growth model with time effect on the probabilities of the *K* attributes *p*(α_*k*_|*time*) as

(9) $$\text{logit}\,p\,\left(\alpha_{k}|t\,i\,m\,e\right)=b_{k}+\gamma_{k}\,\left(t\,i\,m\,e\right)+\zeta_{k}+\varepsilon_{t\,k},\varepsilon_{t\,k}∼N\,\left(0,1\right)$$

Equation 9 represents time is conditioned on the attributes probability, which can be viewed as a predictor of the attribute patterns. Following the interpretation of the RDINA model with covariate extension, *b*_*k*_ represents the fixed-effect attributes difficulty parameter. ζ_*k*_ represents the random intercept parameter for attribute *k*, which allows estimation for each attribute, accounting for individual examinee differences at baseline. Similarly, γ_*k*_ represents the random slope parameter for attribute *k*, which allows differences in examinee rates of growth. ε_*tk*_ represents time-specific error for attribute *k*. Eq. 9.1 shows the associated equation for the random intercept that follows a normal distribution with mean μ_*0k*_ and variance of σ_*0k*_. Eq. 9.2 shows the association equation for the random slope that also follows a normal distribution with mean μ_*1ki*_ and variance of σ_*1k*_. *e*_*0k*_ and *e*_*1k*_ represent the deviations from mean initial level and mean rate of change for attribute *k*, which are random-effect parameters introduced by the latent growth curve model. In this study, the mean and variance of both random effects are fixed to 0 and 1, respectively.

(9.1) $$\zeta_{k}=\eta_{0\,k}=\mu_{0\,k}+e_{0\,k}$$

(9.2) $$\gamma_{k}=\eta_{1\,k}=\mu_{1\,k}+e_{1\,k}$$

Where η_*0*_ and η_*1*_ represent latent trajectory parameters: individual’s initial level and rate of change over time, which was specified in the latent growth curve model framework.

#### Conditional LG-CDM

In addition, we also propose a Conditional LG-CDM based on the conditional latent growth curve model with random intercept and random slope to evaluate the effects of covariate (e.g., intervention effect) on changes in the attribute level. In particular, covariate vector *Z*, is introduced into the latent growth curve model effecting on the random effects. *Z* could be either discrete or continuous covariate. Equation (10) represents the latent growth model with both time and covariate effects on the attribute probability $p\,\left(\alpha_{k}|t\,i\,m\,e,\underline{Z}\right)$ as

(10) $$\begin{array}{c} \text{logit}\,p\,\left(\alpha_{k}|t\,i\,m\,e,\underline{Z}\right)=b_{k}+\gamma_{k}\,\left(t\,i\,m\,e\right)+h_{k}\,\left(\underline{Z}\right)+\zeta_{k}+\varepsilon_{t\,k},\\ \varepsilon_{t\,k}∼N\,\left(0,1\right) \end{array}$$

The interpretation of Conditional LG-CDM is similar to the Unconditional LG-CDM that *b*_*k*_ represents the attributes difficulty parameter, ζ_*k*_ represents the random intercept parameter, and γ_*k*_ represents the random slope parameter. Both random effects follow the normal distribution with fixed mean of 0 and variance of 1 for model identification. ε_*tk*_ represents time-specific error for attribute *k*. What’s more, the parameter *h*_*k*_ represents the regression coefficient of the covariate vector Z, which reflects the shift in the attribute difficulty *b*_*k*_, random intercept ζ_*k*_ and random slope γ_*k*_ when the covariate is present to affect the attribute. Specifically, Eq. 10.1 shows the term for the respective covariate coefficient (*h*_0*k*_) affecting random intercept parameter for each attribute k. Equation 10.2 shows the term for the respective covariate coefficient (*h*_1*k*_) affecting random slope parameter for each attribute k.

(10.1) $$\zeta_{k}=\eta_{0\,k}+h_{0\,k}\,\left(\underline{Z}\right)$$

(10.2) $$\gamma_{k}=\eta_{1\,k}+h_{1\,k}\,\left(\underline{Z}\right)$$

Likewise, η_*0k*_ and η_*1k*_ represent attribute’s initial level and rate of change, which can be extended as shown in Eqs 8.1 and 8.2.

From the perspective of multilevel model, Unconditional LG-CDM and Conditional LG-CDM can be viewed as three-level model where the first level is the time level that involves multiple repeated measures of the same examinee (shown in Eqs 9.1 and 9.2; Eqs 10.1 and 10.2). The second level is the item at individual level that the RDINA model was used to specify an examinee i’s item response (shown in Eq. 1). And the third level is the attribute at individual level that the latent growth curve model was used to specify the change of attributes over time and the covariate effect (Eqs 9 and 10).

In addition, time effect could be specified at the item level as well. Modeled with the RDINA, we let *Y*_*ijt*_ be an examinee *i*’s response for item *j* at time *t*, given the binary latent variable η_*ijt*_ and time. The response probability of a person *i* getting item *j* correctly at time *t* is shown as following:

$$\text{logit}p\left(Y_{i\,j\,t}=1|\eta_{ijt,}time\right)=f_{j}+d_{j}\eta_{i\,j\,t}+\rho_{j}\left(time\right)+{\text{ξ}}_{i\,j\,t}+\varphi_{i\,j\,t}$$

(11.1) $${\text{ξ}}_{i\,j\,t}=\eta_{0\,i}=\mu_{0}+e_{0\,i}$$

(11.2) $$\rho_{j}=\eta_{1\,i}=\mu_{1}+e_{1\,i}$$

where _*ξ ijt*_ represents the random intercept parameter that allows variation in baseline for item response probability *p*(*Y*_*ijt*_ = 1|η_*ijt*,_ time). The ρ_*j*_ represents the random slope parameter that allows growth rate of item response probability vary across time. φ_*ijt*_ is the error term.

Taken together, this study focuses on applying the latent growth curve model into the attribute-level of CDM to analyze how the attributes mastery change over time. There are several advantages to using the Unconditional LG-CDM and Conditional LG-CDM, including that they directly estimate the learning trajectory parameters on the attribute level but also allow covariates effects involved to estimate the intervention effects simultaneously.

## Real-World Data Analysis

### Methods

Real-world data analysis was conducted to motivate the potential of the LG-CDMs and demonstrate its application. We used the pretest and posttest data of a mathematics test (*N* = 879) in the real-world analysis. The mathematic test was developed in a large scale education study that investigated the difficulties for the disabled students in solving mathematic problems (Bottge et al., 2014, 2015). Specifically, an instructional method of Enhanced Anchored Instruction (EAI) was employed in the study to help improve the mathematics achievement of disabled students (Bottge et al., 2003). The design of cluster-randomized controlled trial was used to assign clusters of middle school students to a treatment group or a control group. In the treatment group, students received EAI video sessions about mathematics problem-solving and in the control group, students received instruction method as usual. To evaluate effectiveness of EAI instructional method, the mathematics test was administrated before and after the instructional period and the assessment data was collected. Thus, the dataset consisted of 21-item responses to the test measured four attributes over two time points. A Q-matrix was identified for the four attributes that corresponded to the four instructional units, including α_1_: ratios and proportional relationships, α_2_: measurement and data, α_3_: number systems (fractions), and α_4_: geometry (graphing). Each test item was mapped to one attribute (Appendix). Two latent growth curve CDMs were fit, Unconditional LG-CDM and Conditional LG-CDM.

Data analyses were conducted using Latent GOLD 5.0 (Vermunt and Magidson, 2013).

Two statistics were used to examine attribute classification – (1) proportion correctly classified (*P*_*c*_) and (2) Lambda (λ) (Clogg, 1995), where both are based on the maximum posterior probability to examine the quality of classification. In particular, Eq. (12) shows the use of estimated posterior probabilities in obtaining an estimate of the expected proportion of cases correctly classified for attribute *k* (α_*k*_):

(12) $$P_{c}=\sum_{s}\left[n_{s}\times \text{max}\,p\,\left(\alpha_{k}|\underline{Y_{i\,1}},\underline{Y_{i\,2}},\ldots ,\underline{Y_{i\,J}}\right)\right]/N$$

where *s* represents each unique response pattern, *n*_*s*_ is the frequency of each pattern (i.e., number of cases with a particular pattern), $\text{max}\,p\,\left(\alpha_{k}|\underline{Y_{i\,1}},\underline{Y_{i\,2}},\ldots ,\underline{Y_{i\,J}}\right)$ represents the maximum posterior probability for a given response pattern vector $\left(\underline{Y_{i\,1}},\underline{Y_{i\,2}},\ldots ,\underline{Y_{i\,J}}\right)$, *N* is the total number of cases in a latent class response pattern.

λ makes a correction for classification that can occur by chance, which can be expressed as

(13) $$\lambda =\frac{P_{c}-\text{max}\,p\,\left(\alpha_{k}\right)}{1-\text{max}\,p\,\left(\alpha_{k}\right)}$$

where *p*(α_*k*_) represents the latent class size with *p*(α_*k*_) > 0. Meanwhile, λ also reflects the relative reduction in classification error (Kruskal and Goodman, 1954; Clogg, 1995).

Both expectation-maximization (EM) and Newton-Raphson algorithms were used to obtain maximum likelihood (ML) or posterior mode (PM) estimates. The PM estimation uses a prior distribution to smooth solutions that are near the boundary of the parameter space. Therefore, this method can avoid a boundary estimation issues that are commonly associated with latent class models (DeCarlo, 2011). In addition, to avoid problems of local maxima, 100 sets of starting values were used to obtain the global maximum. Finally, to check for local identification, the rank of the Jacobian matrix was examined to be of full rank as specified as a required condition for local identification in latent class regression models (Huang and Bandeen-Roche, 2004).

### Results

#### Model Fit and Classification

Real-world data converged successfully for the two models, unconditional latent growth curve CDM (Unconditional LG-CDM) and conditional latent growth curve CDM (Conditional LG-CDM). Table 1 shows the classification results. The results show that the *P*_*c*_ estimates are the same for the two LG-CDMs across four mathematics attributes, indicating a satisfactory classification as all statistics are greater than 0.91. For example, with *P*_*c*_ of 0.96 for attribute 2, one would expect that 96% of the cases would be correctly classified into attribute 2. However, it should be noted that if simply classifies all the cases into the attribute with the largest size, then the correctly classification can be achieved but may due to the chance. λ provides a correction for this situation when calculating the proportion correctly classified. The results show that λ are similar for the four attributes across two models and are slightly lower than P_*c*_. For example, the λ classification on attribute 1 in Conditional LG-CDM is 0.70, indicating that the proportion correctly classified increase 70% by using the observers’ response pattern over simply classifying all cases into the attribute with the largest size.

**TABLE 1:** Classification: proportion correctly classified (*P*_*c*_) and Lambda (*λ*).

**Models**	**Classification**	**Attributes**			
		**α_1_**	**α_2_**	**α_3_**	**α_4_**
Unconditional LG-CDM	*P*_*c*_	0.91	0.96	0.92	0.93
	λ	0.79	0.90	0.84	0.82
Conditional LG-CDM	*P*_*c*_	0.91	0.96	0.92	0.93
	λ	0.70	0.90	0.84	0.82

*Classification statistics based on Clogg (1995). Conditional LG-CDM considered EAI instruction. Attributes as follows: α_1_: Ratios and Proportional Relationships; α_2_: Measurement and Data; α_3_: Number Systems (Fractions); α_4_: Geometry (Graphing).*

#### Attribute Prevalence

Table 2 shows the attribute prevalence for the four attributes. The attribute prevalence represents the latent class sizes of the four attributes (DeCarlo, 2011). Overall, the attribute prevalence was consistent across the two models. The probabilities of all attributes prevalence are above 0.50, indicating that more than half of the students mastered each of the attribute. The attribute prevalence for Attribute 2 (Measurement and Data) and Attribute 4 (Geometry) had slightly higher probabilities than the attribute prevalence for Attribute 1 (Ratios and Proportional Relationships) and Attribute 3 (Number Systems).

**TABLE 2:** Attribute prevalence.

**Model**	**α_1_**	**α_2_**	**α_3_**	**α_4_**
Unconditional LG-CDM	0.57 (0.03)	0.62 (0.02)	0.53 (0.03)	0.62 (0.08)
Conditional LG-CDM	0.57 (0.03)	0.62 (0.02)	0.53 (0.03)	0.62 (0.02)

*Values in parenthesis are standard errors. Attributes based on the EAI instruction are as follows: α_1_: Ratios and Proportional Relationships; α_2_: Measurement and Data; α_3_: Number Systems (Fractions); α_4_: Geometry (Graphing).*

#### Item Parameters

Table 3 shows the item parameters (*f*_*j*_ and *d*_*j*_) for the Unconditional LG-CDM and Conditional LG-CDM, which are estimated from the RDINA model. Calculating the exponential of the *f*_*j*_ and *d*_*j*_ parameters, one can obtain guessing and slip parameters of the DINA model (shown in Eqs 1.1 and 1.2). Overall, the item parameters are consistent across the two models. Derived from Eqs 1.1 and 1.2, the average guessing and slip parameters estimates for Unconditional LG-CDM were 0.22 and 0.35; for Conditional LG-CDM were 0.22 and 0.36. With respect to each attribute, the average guessing and slip parameters were respectively the same across the two models: for the attribute of Ratios and Proportional Relationships were 0.14 and 0.49; for the attribute of Measurement and Data were 0.28 and 0.23; for the attribute of Number Systems (Fractions) were 0.28 and 0.23; for the attribute of Geometry (Graphing) were 0.25 and 0.36. In particular, for both Unconditional LG-CDM and Conditional LG-CDM, Item 2 (Attribute of Measurement and Data) has the greatest guessing estimates while Item 17 (Attribute of Ratios and Proportional Relationships) has the greatest slip estimates.

**TABLE 3:** Item parameters for Unconditional and Conditional LG-CDM.

		**Unconditional LG-CDM**		**Conditional LG-CDM**	
**Item**	**Attributes**	***f*_*j*_**	***d*_*j*_**	***f*_*j*_**	***d*_*j*_**
*Y*_1_	α_1_	−2.09(0.02)	1.97 (0.17)	−2.10(0.16)	1.98 (0.17)
*Y*_2_	α_2_	−0.24(0.08)	2.06 (0.13)	−0.24(0.08)	2.06 (0.13)
*Y*_3_	α_2_	−2.61(0.17)	1.92 (0.19)	−2.62(0.18)	1.92 (0.19)
*Y*_4_	α_3_	−1.87(0.13)	1.87 (0.14)	−1.88(0.13)	1.87 (0.14)
*Y*_5_	α_3_	−1.14(0.11)	2.34 (0.13)	−1.14(0.11)	2.34 (0.13)
*Y*_6_	α_3_	−0.74(0.09)	1.88 (0.12)	−0.74(0.09)	1.89 (0.12)
*Y*_7_	α_3_	−3.73(0.42)	3.57 (0.41)	−3.75(0.43)	3.58 (0.42)
*Y*_8_	α_3_	−0.96(0.09)	1.14 (0.12)	−0.96(0.09)	1.14 (0.12)
*Y*_9_	α_2_	−0.76(0.09)	2.21 (0.12)	−0.76(0.09)	2.21 (0.12)
*Y*_10_	α_2_	−0.97(0.10)	2.84 (0.13)	−0.97(0.10)	2.84 (0.13)
*Y*_11_	α_2_	−0.59(0.09)	3.09 (0.15)	−0.59(0.09)	3.09 (0.15)
*Y*_12_	α_2_	−1.38(0.11)	3.03 (0.14)	−1.38(0.11)	3.02 (0.14)
*Y*_13_	α_1_	−0.64(0.10)	2.01 (0.13)	−0.63(0.10)	1.99 (0.13)
*Y*_14_	α_1_	−2.61(0.23)	2.93 (0.22)	−2.60(0.22)	2.91 (0.22)
*Y*_15_	α_4_	−0.99(0.10)	1.91 (0.13)	−0.99(0.10)	1.91 (0.13)
*Y*_16_	α_4_	−0.70(0.10)	1.82 (0.12)	−0.70(0.10)	1.82 (0.12)
*Y*_17_	α_1_	−3.41(0.27)	1.94 (0.29)	−3.49(0.28)	2.02 (0.30)
*Y*_18_	α_4_	−0.27(0.09)	2.35 (0.14)	−0.27(0.09)	2.35 (0.14)
*Y*_19_	α_4_	−1.77(0.13)	1.96 (0.15)	−1.76(0.13)	1.96 (0.15)
*Y*_20_	α_4_	−1.41(0.11)	1.47 (0.13)	−1.41(0.11)	1.47 (0.13)
*Y*_21_	α_4_	−1.92(0.14)	1.63 (0.15)	−1.93(0.14)	1.64 (0.15)

*Values in parenthesis are standard errors. Attributes based on the EAI instruction are as follows: *α*_1_: Ratios and Proportional Relationships; *α*_2_: Measurement and Data; *α*_3_: Number Systems (Fractions); *α*_4_: Geometry (Graphing).*

#### Attribute Parameters and Growth

Table 4 summarized the attribute-level parameters for the Unconditional LG-CDM and Conditional LG-CDM, which include attribute difficulty (*b*_*k*_), intercept and slope of latent growth curve (η_*0*_ and η_*1*_), and regression coefficients (*h*_*k*_) for the intervention effect. In general, the estimates of random intercept and random slope were very close, indicating that the examinees’ initial level and growth rate are similar for the two models. As the Conditional LG-CDM incorporates covariate in addition to the Unconditional LG-CDM, regression coefficients (*h*_*k*_) of the covariate indicate shifts in the attributes difficulty as a result of the treatment effect (EAI). Results show that the parameter estimates of four attributes difficulty are all lower in the Conditional LG-CDM than the Unconditional LG-CDM, suggesting that all attributes were easier to be mastered when involving the treatment effect. In particular, Attribute 1 (Ratios and Proportional Relationships) and Attribute 4 (Geometry) yielded greater differences between the two models then Attribute 2 (Measurement and Data) and Attribute 3 (Number Systems), with a difference of 0.34 and 0.37 units. While most treatment effects were not significant, Attribute 4 (Geometry) had significant treatment effect, shifting the difficulty parameter by 0.24 units.

**TABLE 4:** Attribute difficulty, growth curve and intervention effect parameters.

	**Unconditional LG-CDM**		**Conditional LG-CDM**	
**Parameter**	**Estimate**	**p value**	**Estimate**	**p value**
η_*0*_ (Random Intercept)	0.44 (0.11)		0.46 (0.15)	
η_*1*_ (Random Slope)	0.04 (0.00)		0.03 (0.00)	
*h*_*0k*_ (Treatment Effect on random intercept)	–	–	0.30 (0.19)	0.13
*h*_*1k*_ (Treatment Effect on random slope)	–	–	0.09 (0.00)	<0.001
*b*_1_ (Attribute 1 Difficulty)	−0.11(0.09)		−0.45(0.11)	
*h*_*1*_ (Treatment Effect for Attribute 1)	–	–	0.40 (0.27)	0.13
*b*_2_ (Attribute 2 Difficulty)	0.16 (0.07)		−0.04(0.09)	
*h*_*2*_ (Treatment Effect for Attribute 2)	–	–	0.09 (.25)	0.72
*b*_3_ (Attribute 3 Difficulty)	−0.23(0.08)		−0.44(0.10)	
*h*_*3*_ (Treatment Effect for Attribute 3)	–	–	0.02 (0.26)	0.95
*b*_4_ (Attribute 4 Difficulty)	0.16 (0.04)		−0.11(0.04)	
*h*_*4*_ (Treatment Effect for Attribute 4)	–	–	0.24 (0.00)	<0.001

*(1) Parameter *h*_*k*_ indicates shift in the attributes difficulty due to mastery in treatment effect (EAI), based on the Conditional LG-CDM model. (2) Values in parenthesis represent standard errors. (3) Attributes based on the EAI instruction are as follows: *α*_1_: Ratios and Proportional Relationships; *α*_2_: Measurement and Data; *α*_3_: Number Systems (Fractions); *α*_4_: Geometry (Graphing).*

## Simulation Study I

### Methods

Simulation studies were conducted to evaluate parameter recovery and classification of the two models: (a) Unconditional LG-CDM; (b) Conditional LG-CDM. In simulation study 1, the EAI real-world results were used as generating population (true) values. Following the data structure of the real-world data example, the 21-item response data were generated for two time points; four attributes and Q-matrix were specified as well. Two sample size of 1000 and 2000 were examined across a specification of the four attributes. Therefore, the simulation study includes a total of four simulation conditions (= 2 models × 2 sample size conditions).

Data were generated and fit using Latent GOLD 5.0 (Vermunt and Magidson, 2013). One hundred replications were fitted for each condition. Parameters were estimated using PM estimation and the parameter recovery were evaluated for each condition using three measures: (a) Bias, (b) % Bias, and (c) mean square error (MSE). Here, $B\,i\,a\,s\,\left(x\right)=\frac{1}{N}\,\sum_{n=1}^{N}\left[\hat{e_{n}}\,\left(x\right)-e\,\left(x\right)\right]$, %*Bias* = |*Bias*(*x*)/*e*(*x*)|×100%, $M\,S\,E\,\left(x\right)=\frac{1}{N}\,\sum_{n=1}^{N}{\left[\hat{e_{n}}\,\left(x\right)-e\,\left(x\right)\right]}^{2}$, where *x* is an arbitrary indicator of a parameter, e(x) is the generating (true) parameter value, and $\hat{e_{n}}\,\left(x\right)$ is the *n*th replicate estimate of parameter *x* among a total of *N* = 100 replications. Similar to the real-world data analysis, we set up 100 starting values, and the Jacobian matrix was examined to be of full rank for local identification. EM (expectation-maximization) and Newton-Raphson algorithms were used to avoid a boundary estimation issue using PM estimation. The syntax of unconditional and conditional models is available from the authors upon request.

### Results

#### Parameter Recovery

Table 5 shows the parameter recovery results (Bias, % Bias, and MSE) by sample size (1,000 and 2,000) for the two models. Overall, the parameter recovery revealed consistent estimates. Percent bias for item-level parameters were very close in the two models except item discrimination parameter *dj* was slightly higher in the Conditional LG-CDM model. The parameter *dj* provides a measure of how well the item can discriminate an examinee with or without the mastery of required skills. The overall % bias associated with random effects were all less than 2.0% regardless of models. In Conditional LG-CDM, % bias of random effects were lower (≤1.0%). The intervention effect on attributes in the conditional model had % bias of 37.4% when sample size is 1000. However, it dramatically dropped to 4% when the sample size increase to 2000. The % bias of attribute difficulty parameter are lower in the Unconditional LG-CDM model (6.7 and 3% at the sample sizes of 1,000 and 2,000).

**TABLE 5:** Simulation I results: parameter recovery.

**Model**	**Level**	**Parameter**	**Sample *n* = 1,000**			**Sample *n* = 2,000**		
			**Bias**	**% Bias**	**MSE**	**Bias**	**% Bias**	**MSE**
Unconditional LG-CDM	Random Effects	*λ*	0.007	1.8%	0.002	–0.002	0.5%	0.001
	Attribute Difficulty	*b*_*k*_	–0.002	6.7%	0.002	0.000	3.1%	0.001
	Item	*f*_*j*_	–0.013	1.6%	0.024	–0.001	0.6%	0.007
		*d*_*j*_	0.015	0.9%	0.030	0.002	0.4%	0.009
Conditional LG-CDM	Random Effects	*λ*	0.001	0.3%	0.001	–0.002	0.4%	0.001
		*h*_*k*_	0.002	0.7%	0.003	–0.003	1.0%	0.004
	Attribute Difficulty	*b*_*k*_	0.000	18.2%	0.022	0.000	16.9%	0.002
	Intervention Effect	*h*_*k*_	0.002	37.4%	0.018	–0.001	4.0%	0.018
	Item	*f*_*j*_	–0.009	1.6%	0.022	–0.002	0.7%	0.007
		*d*_*j*_	0.011	1.5%	0.030	0.003	0.5%	0.010

*Simulation based on 100 data replications.*

#### Classification

Simulation classification indices were summarized in Table 6. The results of the two LG-CDM models agree very much. Both *P*_*c*_ and λ showed excellent classification rates on the four attributes in the two models. The classification index was little influenced by the sample sizes.

**TABLE 6:** Simulation classification I: proportion correctly classified (*P*_*c*_) and Lambda (*λ*).

**Models**	**Classification**	***α*_1_**	***α*_2_**	***α*_3_**	***α*_4_**
Unconditional LG-CDM (*n* = 1,000)	*P*_*c*_	0.91	0.98	0.94	0.94
	*λ*	0.80	0.95	0.87	0.84
Conditional LG-CDM (*n* = 1,000)	*P*_*c*_	0.91	0.98	0.94	0.94
	*λ*	0.80	0.94	0.87	0.84
Unconditional LG-CDM (*n* = 2,000)	*P*_*c*_	0.97	0.99	0.98	0.98
	*λ*	0.93	0.99	0.97	0.96
Conditional LG-CDM (*n* = 2,000)	*P*_*c*_	0.97	0.99	0.98	0.98
	*λ*	0.93	0.99	0.97	0.96

*Classification statistics based on Clogg (1995).*

#### Cross Fitting of Simulated Data to Other Models

What’s more, a cross fitting analysis was conducted to examine the consequences on parameter estimates and classification on fitting incorrect models. Data were generated using the Unconditional LG**-**CDM and Conditional LG-CDM and fit with incorrect models one-off, including RDINA model and RDINA model with covariate. The RDINA and RDINA with covariate models do not have a longitudinal component, thereby providing a relative comparison for ignoring the longitudinal component to the model. Model fit indices, *P*_*c*_ and % bias of parameters (random effects, intervention effect, item, and attribute) are presented in the Table 7. All the statistics selected the correct models. For the sample size of 2000, the correct models had higher *P*_*c*_, lower AIC/BIC value as well as lower % bias in both item and attribute parameter estimates. Meanwhile, when fitting with the incorrect models, lower *P*_*c*_, higher AIC/BIC and higher % bias were shown in the outputs. The greatest impact of fitting incorrect models was found in the % bias of attribute and item parameters. When using Conditional LG-CDM generated data and fit with the RDINA with covariates, % bias was more than 101% for the item parameter and attribute difficulty parameter. Similarly, when fitting generated data with RDINA, % bias were also high for the attribute parameter. It is noticeable when data generated using Conditional LG-CDM were fit using Unconditional LG-CDM, % bias are lower than the incorrect RDINA and RDINA with covariate model.

**TABLE 7:** Cross fitting of simulated data.

**Data fit**	**Statistics**	**Data generating conditions (Sample *n* = 2,000)**	
		**Unconditional LG-CDM**	**Conditional LG-CDM**
RDINA	AIC	98964.26	98945.77
	BIC	99234.79	99235.29
	*P*_*c*_	0.87	0.87
	Attribute Difficulty (% Bias)	91.1%	88.4%
	Item (% Bias)	28.1%	28.0%
RDINA with covariates	AIC	–	98847.14
	BIC	–	99161.84
	*P*_*c*_	–	0.87
	Attribute Difficulty (% Bias)	–	101.1%
	Intervention Effect (% Bias)	–	64.5%
	Item (% Bias)	–	101.2%
Unconditional LG-CDM	AIC	**91921.67**	98728.2
	BIC	**92157.24**	98997.04
	*P*_*c*_	**0.98**	0.97
	Random Effects (% Bias)	**0.5%**	13.3%
	Attribute Difficulty (% Bias)	**3.1%**	46.1%
	Item (% Bias)	**0.5%**	1.1%
Conditional LG-CDM	AIC	–	**91971.63**
	BIC	–	**92236.65**
	*P*_*c*_	–	**0.98**
	Random Effects (% Bias)	–	**0.7%**
	Attribute Difficulty (% Bias)	–	**16.9%**
	Intervention Effect (% Bias)	–	**4.0%**
	Item (% Bias)	–	**0.6%**

*Results of the correct model fit were bolded.*

## Simulation Study II

### Methods

We conducted an additional simulation study, where three time points were simulated to examine parameter recovery and classification of the proposed Unconditional and Conditional LG-CDMs. As the data of simulation study I were generated using population (true) values derived from the empirical data that was limited to two time points, the condition was expanded to include more time points in simulation study II so that the potentials of the Unconditional and Conditional LG-CDMs can be fully discussed. In simulation study II, to generate data, we referred to the simulation study design conducted by De La Torre and Douglas (2004) to specify the Q-matrix and item parameters, as generating population (true) value: 30 items with five attributes and 1,000 examinees were used across 100 replications. Table 8 shows the transposed Q-matrix that each attribute appears alone, in pair, or in a triple the same number of times as other attributes. Similar to Simulation Study I, 100 data replications were generated and fit using Latent GOLD 5.0 (Vermunt and Magidson, 2013). Parameters were estimated using PM full name estimation and the parameter recovery were evaluated for each condition using three measures: (a) Bias, (b) % Bias, and (c) mean square error (MSE).

**TABLE 8:** The transposed Q-matrix for the simulation study II.

**Item**															
**Attribute**	**1**	**2**	**3**	**4**	**5**	**6**	**7**	**8**	**9**	**10**	**11**	**12**	**13**	**14**	**15**
1	1	0	0	0	0	1	0	0	0	0	1	1	1	1	0
2	0	1	0	0	0	0	1	0	0	0	1	0	0	0	1
3	0	0	1	0	0	0	0	1	0	0	0	1	0	0	1
4	0	0	0	1	0	0	0	0	1	0	0	0	1	0	0
5	0	0	0	0	1	0	0	0	0	1	0	0	0	1	0

**Attribute**	**16**	**17**	**18**	**19**	**20**	**21**	**22**	**23**	**24**	**25**	**26**	**27**	**28**	**29**	**30**

1	0	0	0	0	0	1	1	1	1	1	1	0	0	0	0
2	1	1	0	0	0	1	1	1	0	0	0	1	1	1	0
3	0	0	1	1	0	1	0	0	1	1	0	1	1	0	1
4	1	0	1	0	1	0	1	0	1	0	1	1	0	1	1
5	0	1	0	1	1	0	0	1	0	1	1	0	1	1	1

### Results

#### Parameter Recovery

Table 9 shows the parameter recovery results of three time points simulation by sample size 1,000 for the two models. Bias, % bias and MSE were lower for item level parameters (discrimination parameter *d*_*j*_ and false rate parameter *f*_*j*_) in the two models, indicating model estimates are consistent in the item level. The results of parameter recovery for attribute difficulty are slightly high as well as for the intervention effect on attribute difficulty. Furthermore, the bias and % bias of random effects (random intercept and random slope) are noticeably high in both models, probably because more time points are involved in the simulation study that ask for more estimations to reach consistent recovery.

**TABLE 9:** Simulation II results: parameter recovery.

**Model**	**Level**	**Parameter**	**Sample *n* = 1,000**		
			**Bias**	**% Bias**	**MSE**
Unconditional LG-CDM	Random Intercept	*λ_*o*_*	–0.592	118.4%	0.354
	Random Slope	*λ_1_*	0.312	62.4%	0.098
	Attribute Difficulty	*b*_*k*_	–0.311	39.0%	0.123
	Item	*f*_*j*_	0.073	5.1%	0.059
		*d*_*j*_	–0.046	2.7%	0.088
Conditional LG-CDM	Random Intercept	*λ_*o*_*	–0.548	109.6%	0.304
		*h*_0k_	0.035	11.8%	0.007
	Random Slope	*λ_1_*	0.315	63.1%	0.101
		*h*_1k_	–0.222	74.1%	0.053
	Attribute Difficulty	*b*_*k*_	–0.297	37.7%	0.122
	Intervention Effect	*h*_*k*_	0.001	20.6%	0.173
	Item	*f*_*j*_	0.065	4.7%	0.058
		*d*_*j*_	–0.038	2.5%	0.082

*Simulation based on 100 data replications.*

#### Classification

Table 10 summarized classification results of three time points simulation. For both LG-CDMs, *P*_*c*_ estimates suggested satisfactory proportion of cases are correctly classified for all attributes (*P*_*c*_ > 0.85). Yet, it should be noted that the classification rates of λ were lower on the second and fourth attributes compared to the other attributes, which may due to the over correction for classification error.

**TABLE 10:** Simulation classification II: proportion correctly classified (*P*_*c*_) and lambda (*λ*).

**Models**	**Classification**	***α*_1_**	***α*_2_**	***α*_3_**	***α*_4_**	***α_5_***
Unconditional LG-CDM (*n* = 1,000)	*P*_*c*_	0.89	0.86	0.98	0.96	0.95
	*λ*	0.72	0.47	0.79	0.36	0.76
Conditional LG-CDM (*n* = 1,000)	*P*_*c*_	0.90	0.88	0.98	0.97	0.94
	*λ*	0.76	0.50	0.81	0.35	0.81

*Classification statistics based on Clogg (1995).*

## Discussion and Conclusion

Cognitive diagnostic models have become increasingly important in educational measurement by estimating skill profiles that indicate the examinee’s mastery in fine-grained skills based on their performance (Rupp et al., 2010). In most prior studies, CDMs have been applied to single cross-sectional time diagnosis instead of tracking the changes in skills or attributes. However, learning is a process during which students acquire knowledge and improve their skills. As learning progress, students’ skills mastery and knowledge could change over time. In addition, the implementation of particular intervention may influence students’ learning trajectory, which is important for educators to know in order to evaluate learning and instruction. In this study, we propose two latent growth CDMs, Unconditional LG-CDM and Conditional LG-CDM, to assess students’ change in skills mastery over time and evaluate the intervention effect on the growth of skill mastery.

Results from the real-world data analysis showed that the latent growth curve model and covariate extension such as intervention effect, could be used to link with a CDM. The statistics of model classification and attribute prevalence agree very much and are excellent for the two LG-CDMs, indicating both models are well specified to provide consistency results. In particular, although the latent class size of Attribute 1 and Attribute 3 are slightly lower than Attribute 2 and Attribute 4, more than half of the students mastered each attribute. Moreover, results showed that the estimates of attribute difficulty of the Conditional LG-CDM was generally lower than the estimates of the Unconditional LG-CDM. The decrease in attribute difficulty indicated that the attributes have been shifted and implies that it become easier for students to master the attributes when involving educational intervention, which was further confirmed by the results of growth curve and intervention effect parameters. Both the Unconditional LG-CDM and Conditional LG-CDM examined the students’ performance at baseline and the growth rate. Although the baseline performance is slightly different, the grow rates are similar across the two models. Thus, the LG-CDMs could inform the researchers and educators that the EAI method has little effect on the growth rate of student’ ability. However, with the help of Conditional LG-CDM that incorporate the covariates extension into CDM, we can tell that the treatment EAI method does improve students’ mastery on the attribute 1 and attribute 4. In other words, if the students were assigned to the treatment group that receiving the EAI teaching method, they would show progress in math learning, especially in the skills of geometry and ratio/proportional relationships.

The simulation studies showed that the parameters were consistently recovered in general, indicating that incorporating latent growth curve and covariate extension at the attribute level did not affect model estimation. In simulation study I, both attribute and item level estimates were stable with sample size of 1,000 and 2,000 for the Unconditional LG-CDM. For the Conditional LG-CDM, additional attention may be given to the attribute difficulty parameter (*b*_*k*_) and intervention effect parameter (*h*_*k*_), percentage bias of 18.2% and 37.4% for the sample size of 1,000. However, with the sample size increase to 2,000, the % bias of intervention effect declines rapidly to 4%. The recovery of random parameters was excellent across two models, with bias of random intercepts are less than 2.0% in the sample size of 1,000 and 2,000.

In Simulation Study II, more time points and items were involved to fully examine the performance of proposed LG-CDMs in terms of parameter recovery. The item level estimates were satisfactory with sample size of 1,000. Although the bias and % bias of attribute difficulty parameter and intervention effect parameters are slightly high, it is expected that they would decrease obviously when the sample size increase. However, it should be noted that the recovery of random effects for data with three time-point specifications were modest and may depend on study design. Thus, latent growth models may need more specifications or constrains on random intercept and random slope parameters to achieve stable recovery. Besides, the classification indices of the both simulation studies were consistent across different conditions. The results obtained from this study help to advance CDMs to better measuring the change in learning over time.

Researchers and educators have long used pre-post assessment to evaluate the effects of new curriculum and teaching method on students’ learning. Meanwhile, it is important to know students’ learning trajectory to achieve learning goal. Cognitive diagnostic models have provided a diagnostic framework to measure students’ mastery in fine-grained skills and different kinds of longitudinal analysis have been incorporate to the CDM to evaluate changes in skills profile mastery (e.g., Li et al., 2016; Kaya and Leite, 2017). Different from other longitudinal CDMs, the LG-CDMs described in this article incorporate well-established latent growth curve model that is more widely used in the social studies. Additionally, covariate extension was integrated to signify the intervention effect. Dayton and Macready (1988) introduced the use of covariate to affect the attribute. Park et al. (2017) included both observed and latent explanatory variables as covariates in the explanatory CDM to inform learning and practice. Thus, this approach is meaningful in the CDM for its diagnostic purpose. In this study, latent growth curve and covariates are both specified at the attribute level. In addition, latent growth curve could be also specified at the item level, contributing to the multilevel studies on CDMs. Meanwhile, more attributes and covariates could be incorporated in specifying the items and explaining the relationship among them. In the empirical study, it is likely to have items that are of high slip and guessing estimates in the test (Lee et al., 2011), therefore, it is important for the researchers to carefully develop and validate the Q-matrix used for CDM analyses.

For the future studies, different types of variance-covariance structures could be specified in the model. For example, all the parameters could be freely estimated (unstructured) in the covariance structure to explore their relationships in model estimation. Meanwhile, future studies could conduct a comprehensive investigation of the measurement invariance, building on the foundational simulation studies conducted in this paper. For example, additional parameters could be included to examine their effects on model identification and their impact on the measurement invariance. Furthermore, the item level fit statistics could be developed in the future studies for the item-level effects in the LG-CDMs, which provides suitable item level fit information for the studies that involve multiple time points. In the current field of education, individualized learning has been emphasized, which allows students to construct learning progress at own pace. This study provides a flexible framework to diagnose skill mastery as well as advancing the longitudinal CDMs to better measuring the change in learning over time.

## Data Availability Statement

The data analyzed in this study is subject to the following licenses/restrictions: The use of datasets requires permission. Requests to access these datasets should be directed to University of Kentucky.

## Author Contributions

QL contributed to the conceptualization of the manuscript and conducted formal analysis as well as wrote the original draft. KX contributed to the review and edition of the manuscript. YP contributed to the supervision, conceptualization, and review of the manuscript. All authors contributed to the article and approved the submitted version.

## Conflict of Interest

The authors declare that the research was conducted in the absence of any commercial or financial relationships that could be construed as a potential conflict of interest.

## Appendix

**TABLE A1:** The Q-matrix for the real-world data analysis.

	**Attribute**			
**Item**	***α*_1_**	***α*_2_**	***α*_3_**	***α*_4_**
1	1	0	0	0
2	0	1	0	0
3	0	1	0	0
4	0	0	1	0
5	0	0	1	0
6	0	0	1	0
7	0	0	1	0
8	0	0	1	0
9	0	1	0	0
10	0	1	0	0
11	0	1	0	0
12	0	1	0	0
13	1	0	0	0
14	1	0	0	0
15	0	0	0	1
16	0	0	0	1
17	1	0	0	0
18	0	0	0	1
19	0	0	0	1
20	0	0	0	1
21	0	0	0	1
